# Hetero-oligomeric interactions of an ELOVL4 mutant protein: implications in the molecular mechanism of Stargardt-3 macular dystrophy

**Published:** 2010-11-18

**Authors:** Ayaka Okuda, Tatsuro Naganuma, Yusuke Ohno, Kensuke Abe, Maki Yamagata, Yasuyuki Igarashi, Akio Kihara

**Affiliations:** 1 Laboratory of Biochemistry, Faculty of Pharmaceutical Sciences, Hokkaido University, Sapporo, Japan; 2 Laboratory of Biomembrane and Biofunctional Chemistry, Faculty of Advanced Life Science, Hokkaido University, Sapporo, Japan

## Abstract

**Purpose:**

Stargardt disease 3 (STGD3) is a juvenile macular dystrophy caused by mutations in the elongase of very long-chain fatty acids-like 4 (*ELOVL4*) gene, which encodes an elongase involved in the production of extremely long-chain fatty acids. The STGD3-related mutations cause production of C-terminally truncated proteins (ELOVL4ΔC). STGD3 is transmitted in an autosomal dominant manner. To date, molecular mechanisms of this pathology have been proposed based solely on the interaction between wild-type ELOVL4 and ELOVL4ΔC. However, analyses of *Elovl4*Δ*C* knockin mice revealed reduced levels of not only ELOVL4 substrates, but also of fatty acids with a broad spectrum of chain lengths. Therefore, we investigated the molecular mechanisms responsible for ELOVL4ΔC affecting the entire very long-chain fatty acid (VLCFA) elongation pathway.

**Methods:**

The ELOVL4ΔC protein was expressed in HEK 293T cells, and its effect on elongase activities toward several acyl-CoAs were examined. We also investigated the homo- and hetero-oligomerization of ELOVL4ΔC with other elongases (ELOVL1–7) or with other enzymes involved in VLCFA elongation using coimmunoprecipitation experiments.

**Results:**

We found that ELOVL4ΔC forms a homo-oligomer more strongly than wild-type ELOVL4. ELOVL4ΔC also interacts strongly with other elongases, although similar interactions for wild-type ELOVL4 were observed as only weak. In addition, ELOVL4ΔC is able to form an elongase complex by interacting with other components of the VLCFA elongation machinery, similar to wild-type ELOVL4.

**Conclusions:**

We propose that not only the ELOVL4-ELOVL4ΔC homo-oligomeric interaction, but also several hetero-oligomeric interactions, may contribute to the pathology of STGD3.

## Introduction

Stargardt disease 3 (STGD3) is a juvenile-onset macular dystrophy, characterized by gradual loss of central vision, accumulation of lipofuscin, and window defects in the macula [1,2]. STGD3, which is transmitted in an autosomal dominant manner, is caused by mutations in the elongase of very long-chain fatty acids-like 4 (*ELOVL4*) gene, which encodes an elongase involved in the production of extremely long-chain fatty acids (FAs) [3]. The highest expression of *ELOVL4* mRNA has been observed in the retina, followed by the skin, brain, and testis [3,4]. To date, three types of *ELOVL4* mutations have been found in STGD3 patients [2]. All of these mutations result in a C-terminally truncated version (ELOVL4ΔC) of the protein. In addition, as the wild-type protein normally carries an endoplasmic reticulum (ER) retention signal in its C-terminus, all three mutations also cause a loss of the ER retention signal. Subsequently, while wild-type ELOVL4 is localized in the ER, ELOVL4ΔC is mislocalized to the Golgi or aggresomes [5-7]. Furthermore, coexpression of ELOVL4ΔC with wild-type ELOVL4 results in the mislocalization of the wild-type protein due to its interaction with the mutated protein [6-8]. This effect is considered to be the molecular basis for the autosomal dominant transmission of STGD3.

Very long-chain fatty acids (VLCFAs), FAs with a chain length of ≥C20, function in numerous cellular processes, including sphingolipid biogenesis, inflammation, immunity, fetal growth and development, retinal function, and brain development [9-11]. VLCFA elongation occurs in the ER on acyl-CoAs by adding two carbon units in each cycle, and is composed of four steps: condensation, reduction, dehydration, and reduction [10]. The second and fourth reduction steps are catalyzed by the reductases 3-ketoacyl-CoA reductase (KAR) and trans-2,3-enoyl-CoA reductase (TER), respectively [12], while 3-hydroxyacyl-CoA dehydratase (HACD) proteins (HACD1–4) are responsible for the third step, catalyzing the dehydration of 3-hydroxyacyl-CoA [13]. The first step of the VLCFA elongation, condensing malonyl-CoA and acyl-CoA, is rate-limiting, and is catalyzed by one of seven elongases (ELOVL1–7) [10,14]. ELOVL1–7 differ in substrate specificities [10,15]. The substrates of ELOVL4 are predicted to be fatty acyl-CoAs with extremely long chain-lengths (≥C26) [16-18]. Such FAs exist only in certain tissues. Saturated FAs are observed in skin and are used in the formation of ceramides, the major lipid components of stratum corneum. *Elovl4* knockout mice die soon after birth due to defects in skin barrier formation [16]. On the other hand, polyunsaturated, extremely long FAs are found in the retina, sperm, and brain [2].

In addition to the *Elovl4* knockout mice, *Elovl4*Δ*C* knockin mice have been generated and used as a model for STGD3 [17,18]. The phenotype of homozygous knockin mice resembles that of the *Elovl4* knockout mice: They die within a few hours after birth, exhibiting severe defects in skin barrier formation [17,18]. This would suggest that the Elovl4ΔC protein has no enzyme activity. In addition, heterozygous *Elovl4*Δ*C* knockin mice display STGD3-like phenotypes such as progressive photoreceptor degeneration and the accumulation of lipofuscin in the retinal pigment epithelium [19]. Quantitative lipid analyses have demonstrated that the levels of retinal phosphatidylcholines with C32-C36 polyunsaturated FAs (PUFAs) are reduced in the heterozygous *Elovl4*Δ*C* knockin mice [20]. Unexpectedly, though, not only these ELOVL4 products, but also FAs with a broad spectrum of acyl-chain lengths, are affected in retinas by the *Elovl4*Δ*C* mutation [19]. This implies that the entire VLCFA elongation machinery is affected by the Elovl4ΔC protein. In the present study, we determined that the expression of ELOVL4ΔC does indeed result in the inhibition of elongase activities toward C16:0-, C18:0-, C18:3(n-6)-, and C20:4(n-6)-CoAs in vitro. We also found that ELOVL4ΔC interacts with other ELOVLs, in addition to its already established homo-oligomeric interaction with wild-type ELOVL4. Inhibition of the entire VLCFA elongation pathway due to hetero-oligomer formation may contribute, at least partly, to the pathology of STGD3.

## Methods

### Cell culture and transfection

HEK 293T cells were grown on 0.3% collagen-coated dishes in Dulbecco’s modified Eagle’s medium (DMEM; Sigma, St. Louis, MO) containing 10% fetal bovine serum and supplemented with 100 units/ml penicillin and 100 μg/ml streptomycin.

Transfections were performed using Lipofectamine Plus^TM^ Reagent (Invitrogen, Carlsbad, CA) according to the manufacturer’s instructions. Cells were grown to ~60% confluency in six-well dishes, and medium was changed to 0.8 ml OPTI-MEM I Reduced-Serum Medium (Invitrogen) 10 min before transfection. Plasmid DNAs (0.5 μg) were diluted into 100 μl OPTI-MEM I, and 4 μl PLUS reagent was added to the DNA solution. After a 15 min incubation at room temperature, DNA-PLUS solution was mixed with 3 μl Lipofectamine reagent diluted with 100 μl OPTI-MEM I, and the mixture was incubated at room temperature for 15 min. The DNA-PLUS-Lipofectamine reagent complex was then added to cells. After incubation at 37 °C for 3 h, the medium was removed and replaced with normal medium.

### Plasmids

The pCE-puro HA-1 and the pCE-puro His_6_-Myc-1 plasmids are mammalian expression vectors designed to produce an N-terminal HA-tagged protein and an N-terminal tandemly oriented His_6_ and a Myc epitope (His_6_-Myc)-tagged protein, respectively. The pCE-puro HA-ELOVLx (where x is each ELOVL number) plasmids have been described previously [13]. The pCE-puro His_6_-Myc-ELOVL4 plasmid was constructed by cloning the *ELOVL4* gene from the pCE-puro HA-ELOVL4 plasmid into the pCE-puro His_6_-Myc-1 vector.

The *KAR* and *TER* genes were amplified from an expressed sequence tag (EST) clone (ID 3622879; Open Biosystems, Huntsville, AL) and from human liver cDNA (Clontech [TAKARA Bio], Palo Alto, CA), respectively, by PCR using primers (for KAR, 5′-AGG ATC CAT GGA GAG CGC TCT CCC CGC CGC CG-3′ [BamHI site underlined] and 5′-TTT AGT TCT TCT TGG TTT TCT TCA GAT AG-3′; and for TER, 5′-AGG ATC CAT GAA GCA TTA CGA GGT GGA GAT TCT G-3′ [BamHI site underlined] and 5′-TTC AGA GCA GGA AGG GGA TGA TGG GC-3′). The resulting fragments were first cloned into pGEM-T Easy vector (Promega, Madison, WI), generating the pGEM-KAR and pGEM-TER plasmids. The pCE-puro HA-KAR and pCE-puro HA-TER plasmids were constructed by cloning the BamHI-NotI fragments of each of the pGEM-KAR and pGEM-TER plasmids into a pCE-puro HA-1 vector.

The pCE-puro His_6_-Myc-ELOVL4ΔC and pCE-puro HA-ELOVL4ΔC plasmids were constructed as follows. The *ELOVL4* gene with a deletion at its 3′-terminus was amplified by PCR using the primers 5′-AGG ATC CAT GGG GCT CCT GGA CTC GGA GCC GG-3′ (BamHI site underlined) and 5′-CAA TCA GCT TCA TAT TTC TCT TTC TTT AAA-3′ from the pCE-puro HA-ELOVL4 plasmid. The amplified fragment was first cloned into the pGEM-T Easy vector, and the BamHI-NotI fragment of the resulting plasmid was transferred to the pCE-puro HA-1 vector or pCE-puro His_6_-Myc-1 vector, producing the pCE-puro HA-ELOVL4ΔC and pCE-puro His_6_-Myc-ELOVL4ΔC plasmids, respectively.

### In vitro fatty acid elongation assays

In vitro FA elongation assays were performed essentially as described elsewhere [21] using total membrane fractions. Cells were suspended in buffer A (50 mM HEPES-NaOH [pH 6.8], 150 mM NaCl, 10% glycerol, 1× protease inhibitor mixture [Complete^TM^ EDTA free; Roche Diagnostics, Indianapolis, IN], 1 mM PMSF, and 1 mM DTT) and lysed by sonication. After ultracentrifugation (100,000× g, 30 min, 4 °C), the pellet was suspended in buffer A and was used as the total membrane fraction. Total membrane fractions (20 μg protein in 19.5 μl buffer A) were each mixed with 25 μl of 2× assay buffer (300 mM potassium phosphate buffer [pH 6.8], 10% glycerol, 4 mM MgCl_2_, 2 mM CaCl_2_, 2 mM NADPH, 1× protease inhibitor mixture, 1 mM PMSF, and 1 mM DTT), 2.5 μl of 1 mM acyl-CoA (Avanti Polar Lipids, Alabaster, AL) complexed with 4 mg/ml FA-free BSA (Sigma), and 3 μl of 25 μCi/ml [^14^C]malonyl-CoA (55 mCi/mmol; Moravek Biochemicals, Brea, CA). After a 30 min incubation period at 37 °C, the reactions were terminated by adding 25 μl 75% KOH (w/v) and 50 μl ethanol, then saponified at 70 °C for 1 h, and acidified by adding 100 μl of 5 N HCl with 50 μl of ethanol. Lipids were extracted with 750 μl hexane and dried, then suspended in 30 μl chloroform and separated by normal phase thin layer chromatography (TLC) on LK5DF Silica Gel 150A TLC plates (Whatman, Kent, UK) with hexane/diethyl ether/acetic acid (30:70:1, v/v) as the solvent system. Labeled FAs were detected and quantified by a bioimaging analyzer BAS-2500 (Fuji Photo Film, Tokyo, Japan).

When FAs (eicosapentaenoic acid [EPA] or docosapentaenoic acid [DPA]) were used in place of acyl-CoAs, total membrane fractions (20 μg protein in 17.83 μl buffer A) were each mixed with 25 μl of 2× assay buffer, 0.5 μl of 5 mM FAs (Sigma), 1.67 μl of 0.3 M ATP, 2 μl of 5 mM CoA, and 3 μl of 25 μCi/ml [^14^C]malonyl-CoA. After termination of the reactions, lipids were saponified, acidified, extracted, and separated by TLC, followed by detection and quantification by a bioimaging analyzer BAS-2500, as described above.

Statistical analyses were performed by a two-tailed Student *t* test using Microsoft Excel software (Microsoft Corporation, Redmond, WA).

### Immunoblotting

Immunoblotting was performed as described previously [22] using the anti-HA antibody HA-7 (1:2,000 dilution; Sigma), the anti-Myc antibody PL14 (0.5 μg/ml; Medical & Biologic Laboratories, Nagoya, Japan), or the anti-calnexin (H-10) antibody (0.2 μg/ml; Santa Cruz Biotechnology, Inc., Santa Cruz, CA) as the primary antibody and horse radish peroxidase (HRP)-conjugated anti-mouse or anti-rabbit IgG F(ab’)_2_ fragment (each from GE Healthcare Bio-Sciences, Piscataway, NJ, and diluted 1:7500) as the secondary antibody. Labeling was detected using ECL^TM^ Reagents or an ECL plus System for Western Blotting Detection (both from GE Healthcare Bio-Sciences).

### Coimmunoprecipitation

HEK 293T cells were transfected with two plasmids, one carrying HA-tagged genes and the other harboring Myc-tagged genes. Twenty-four hours after transfection, the cells were washed twice with PBS, suspended in buffer B (50 mM HEPES-NaOH (pH 7.4), 150 mM NaCl, 10% glycerol, 1× protease inhibitor mixture, 1 mM PMSF, and 1 mM DTT), and sonicated. After a centrifugation at 300× g for 3 min at 4 °C, the resulting supernatant was treated with 1% Triton X-100 for 30 min at 4 °C, to solubilize membranes. Samples were centrifuged at 100,000× g for 30 min at 4 °C, and the supernatant was incubated overnight at 4 °C with the anti-HA antibody HA-7 conjugated to agarose (Sigma). The gel was washed twice with buffer B containing 0.1% Triton X-100, suspended in 2× sodium dodecyl sulfate (SDS) sample buffer, and incubated for 5 min at 37 °C. The obtained precipitates were separated by SDS–PAGE (PAGE), and subjected to immunoblotting with the HA7 antibody or an anti-Myc PL14 antibody.

## Results

### ELOVL4 exhibits no activity toward eicosapentaenoic acid or docosapentaenoic acid

**Figure 1:**
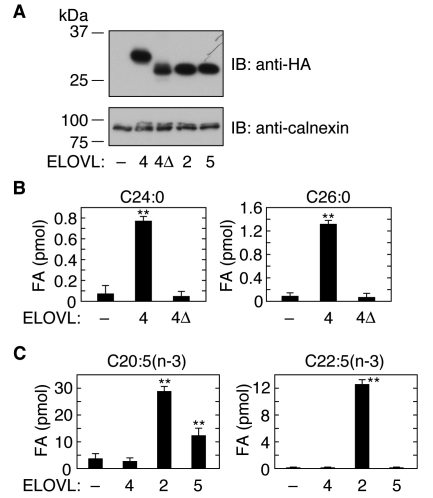
ELOVL4 is not involved in the elongation of eicosapentaenoic acid (EPA) or docosapentaenoic acid (DPA). HEK 293T cells were transfected with a vector (pCE-puro HA-1) or a plasmid encoding the indicated HA-tagged human ELOVL protein. **A**: Total membrane proteins (3 μg protein) prepared from the transfected cells were separated by sodium dodecyl sulfate PAGE (SDS–PAGE), and detected by immunoblotting with anti-HA antibodies. Uniform protein loading was demonstrated by immunoblotting with anti-calnexin antibodies. **B**, **C**: Total membrane proteins (20 μg of protein) were incubated for 30 min at 37 °C with 50 μM acyl-CoA(C24:0-CoA or C26:0-CoA; **B**) or with 50 μM FAs (EPA or DPA), 10 mM ATP, and 200 μM CoA (**C**) in the presence of 1 mM NADPH and 0.075 μCi [^14^C]malonyl-CoA. After termination of the reactions, lipids were saponified, acidified, extracted, and separated by normal phase thin layer chromatography (TLC), followed by detection and quantification by a bioimaging analyzer BAS-2500. Values presented represent the mean±standard deviation (SD) from three independent experiments. Statistically significant differences compared to vector-transfected cells are indicated (**p<0.01; *t*-test). Abbreviations: IB represents immunoblotting, 4 represents ELOVL4; 4Δ represents ELOVL4ΔC; 2 represents ELOVL2; 5 represents ELOVL5.

We recently performed comprehensive in vitro analyses examining substrate specificities of all ELOVLs using [^14^C]malonyl-CoA and 11 acyl-CoAs (C16:0-, C18:0-, C18:1(n-9)-, C18:2(n-6)-, C18:3(n-3)-, C18:3(n-6)-, C20:0-, C20:4(n-6)-, C22:0-, C24:0-, or C26:0-CoA). In those studies ELOVL4 exhibited activity toward C24:0- and C26:0-CoAs, but had no activity toward the other acyl-CoAs [15]. The phenotype of homozygous *Elovl4*Δ*C* knockin mice resembles that of the *Elovl4* knockout mice [17,18], suggesting that the Elovl4ΔC protein has no enzyme activity. However, to date, the enzyme activity of ELOVL4ΔC has not been directly examined. Therefore, we constructed an *ELOVL4*Δ*C* plasmid encoding an HA-tagged mutant ELOVL4 protein, which has a truncation of the C-terminal 51 amino acid residues, and expressed the protein in HEK 293T cells (Figure 1A). We then performed an in vitro FA elongase assay using total membrane proteins prepared from HEK 293T cells overproducing HA-ELOVL4 or HA-ELOVL4ΔC, together with [^14^C]malonyl-CoA and C24:0-CoA or C26:0-CoA. Consistent with our previous results [15], wild-type ELOVL4 exhibited enzyme activity toward both C24:0-CoA and C26:0-CoA (Figure 1B). In contrast, ELOVL4ΔC had no activity toward either substrate.

Reportedly, in cell lines that do not express significant levels of *ELOVL4* mRNA, the expression of Elovl4 and subsequent treatment with the FAs EPA (C20:5(n-3)) or DPA (C22:5(n-3)) resulted in the production of C28 to C38 PUFAs [23]. However, reported results were unclear as to whether these FAs were direct substrates of ELOVL4, since no biochemical analysis was performed. Therefore, we performed an in vitro FA elongase assay using HEK 293T cells overproducing HA-ELOVL4. Since C22:5(n-3)-CoA is not commercially available, we used ATP, CoA, and EPA or DPA, in place of C20:5(n-3)-CoA or C22:5(n-3)-CoA. In this assay system, EPA or DPA is converted to C20:5(n-3)-CoA or C22:5(n-3)-CoA, respectively, by endogenous acyl-CoA synthase using ATP and CoA. For use as controls, HEK 293T cells were also transfected with vector or with a plasmid encoding HA-ELOVL2 or HA-ELOVL5. Immunoblotting demonstrated that ELOVL4 was expressed at levels comparable to those of ELOVL2 and ELOVL5 (Figure 1A). Consistent with previous reports [21,24,25], ELOVL2, and to a lesser extent, ELOVL5, exhibited activities toward EPA, and ELOVL2 was active toward DPA (Figure 1C). However, ELOVL4 had no activity toward either substrate (Figure 1C). These results suggest that neither C20:5(n-3)-CoA nor C22:5(n-3)-CoA is a substrate of ELOVL4.

### Expression of ELOVL4ΔC results in inhibition of other elongation pathways in which ELOVL4 is not directly involved

**Figure 2:**
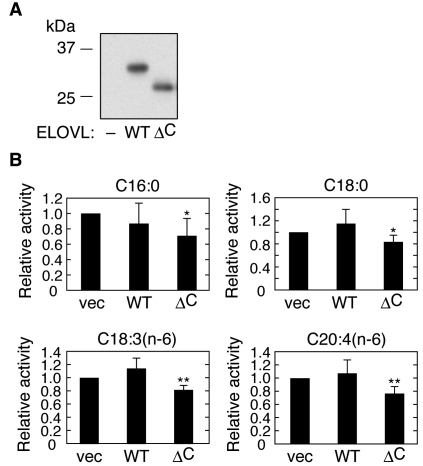
Expression of ELOVL4ΔC reduces endogenous fatty acid (FA) elongation activities. HEK 293T cells were transfected with a pCE-puro HA-1 (vector), pCE-puro HA-ELOVL4, or pCE-puro HA-ELOVL4ΔC plasmid. Forty-eight hours after transfection, total membrane proteins were prepared from the transfected cells. **A**: Total membrane proteins (2 μg of protein) were separated by sodium dodecyl sulfate PAGE (SDS–PAGE), followed by immunoblotting with anti-HA antibodies. **B**: Total membrane proteins (20 μg protein) were incubated with the indicated acyl-CoA (50 μM) and 0.075 μCi [^14^C]malonyl-CoA in the presence of 1 mM NADPH, for 30 min at 37 °C. After termination of the reactions, lipids were saponified, acidified, extracted, and separated by normal phase thin layer chromatography (TLC). The radioactivities associated with the reaction product fatty acid (FAs) were quantified using a bioimaging analyzer BAS-2500. Values shown are relative to those for vector-transfected cells, and represent the mean±standard deviation (SD) from three independent experiments. Statistically significant differences compared to vector-transfected cells are indicated (*p<0.05, **p<0.01; *t*-test). Abbreviations: WT represents wild-type; ΔC represents ELOVL4ΔC; vec represents vector.

Lipid composition analyses determined that in retinas of *Elovl4*Δ*C* knockin mice, not only levels of C32-C36 lipids, the putative direct products of ELOVL4, but also of lipids with a broad range of chain-length, are reduced [19,20]. This suggests that ELOVL4ΔC inhibits elongation reactions that are not catalyzed by ELOVL4. To exclude the possibility that long-term expression of Elovl4ΔC causes indirect metabolic or transcriptional changes in lipid-related genes in the *Elovl4*Δ*C* knockin mice, we introduced ELOVL4ΔC into HEK 293T cells and analyzed its effects on endogenous elongase activities toward C16:0-, C18:0-, C18:3(n-6)-, and C20:4(n-6)-CoAs. In vitro analyses previously revealed the responsible ELOVLs active toward these acyl-CoAs: C16:0-CoA, ELOVL6; C18:0-CoA, ELOVL3 and ELOVL7; C18:3(n-6)-CoA, ELOVL5 and ELOVL7; C20:4(n-6)-CoA, ELOVL2 and ELOVL5 [10,15]. Forty-eight hours after transfection of the cells with vector or a plasmid encoding wild-type ELOVL4 or ELOVL4ΔC, total membrane proteins were prepared. Immunoblotting demonstrated that wild-type ELOVL4 and ELOVL4ΔC were expressed at similar levels (Figure 2A). Expression of wild-type ELOVL4 had no effect on the elongation of C16:0-, C18:0-, C18:3(n-6)-, or C20:4(n-6)-CoAs (Figure 2B), consistent with previous results indicating that these acyl-CoAs are not substrates for ELOVL4 [15]. On the other hand, expression of ELOVL4ΔC resulted in a slight but statistically significant reduction in the products of all the tested elongation reactions (Figure 2B). These results suggest that ELOVL4ΔC inhibits the entire VLCFA elongation pathway.

### ELOVL4ΔC interacts with other ELOVLs

**Figure 3:**
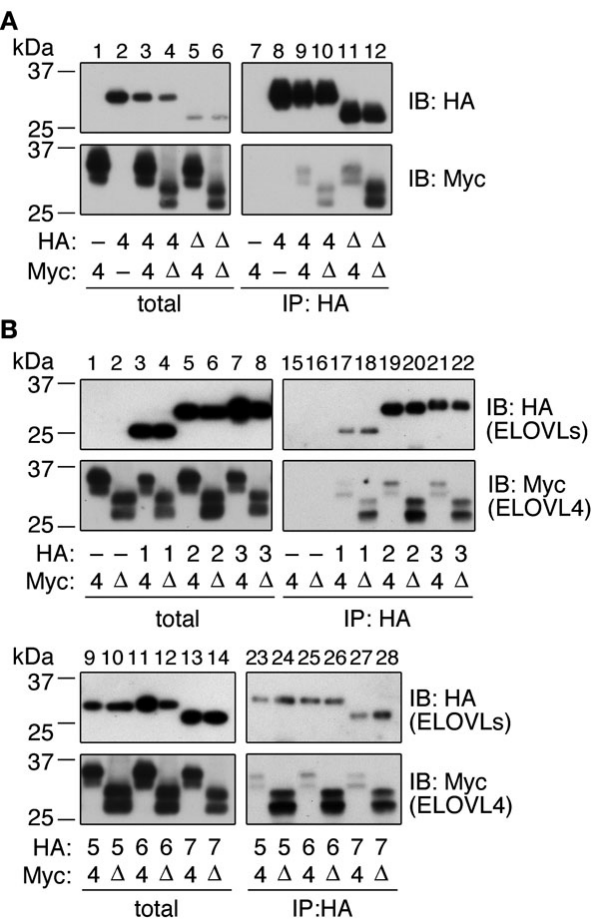
ELOVL4ΔC interacts with other ELOVLs. HEK 293T cells were transfected with a pCE-puro HA-1 (vector; **A** and **B**), pCE-puro HA-ELOVL4 (**A**), pCE-puro HA-ELOVL4ΔC (**A**), or pCE-puro HA-ELOVLx plasmid (where x represents each ELOVL number; **B**), and with the pCE-puro His_6_-Myc-1 (vector; **A**), pCE-puro His_6_-Myc-ELOVL4 (**A** and **B**), or pCE-puro His_6_-Myc-ELOVL4ΔC plasmid (**A** and **B**). Total cell lysates were prepared from the transfected cells and solubilized with 1% Triton X-100. Following immunoprecipitation with the anti-HA antibody, total lysates (1×) and bound proteins (**A**, 8×; **B**, IB: HA, 1×; **B**, IB: Myc, 16.5×) were subjected to immunoblotting with anti-HA or anti-Myc antibodies. IP, immunoprecipitation; IB, immunoblotting; -, vector; 4, wild-type ELOVL4; Δ, ELOVL4ΔC; 1, ELOVL1; 2, ELOVL2; 3, ELOVL3; 5, ELOVL5; 6, ELOVL6; 7, ELOVL7.

The interaction between wild-type ELOVL4 and ELOVL4ΔC has already been established by coimmunoprecipitation, sucrose density gradient sedimentation, and native PAGE [6-8]. To compare the strength of interactions between ELOVL4-ELOVL4, ELOVL4-ELOVL4ΔC, and ELOVL4ΔC-ELOVL4ΔC, we performed coimmunoprecipitation experiments. HA-tagged ELOVL4 or ELOVL4ΔC was expressed in HEK 293T cells together with Myc-tagged ELOVL4 or ELOVL4ΔC, and each was subjected to coimmunoprecipitation with anti-HA antibodies, following membrane solubilization with the nonionic detergent Triton X-100. Myc-tagged ELOVL4 and Myc-tagged ELOVL4ΔC were each detected as two bands, which correspond to their glycosylated and unglycosylated forms [7] (Figure 3A). Expression of wild-type Myc-ELOVL4 alone did not result in its capture by anti-HA agarose beads (Figure 3A, lane 8). However, low levels of wild-type Myc-ELOVL4 were detected in immunoprecipitates with wild-type HA-ELOVL4 (Figure 3A, lane 9), suggesting a weak interaction. Although the Myc-tagged ELOVL4ΔC was expressed at levels lower than those of wild-type Myc-ELOVL4 (Figure 3A, lane 4), higher levels of the Myc-ELOVL4ΔC protein were observed in the immunoprecipitates of wild-type HA-ELOVL4 (Figure 3A, lane 10) than levels of wild-type Myc-ELOVL4 (Figure 3A, lane 9). These results suggest that the C-terminal deletion strengthens the ELOVL4 homointeraction. A similarly enhanced interaction was observed between HA-ELOVL4ΔC and wild-type Myc-ELOVL4 (Figure 3A, lane 11). Moreover, the interaction between HA-ELOVL4ΔC and Myc-ELOVL4ΔC was even stronger than the wild-type ELOVL4-ELOVL4ΔC interaction (Figure 3A, lane 12).

Since the expression of ELOVL4ΔC resulted in the inhibition of other elongation pathways not catalyzed by ELOVL4 (Figure 2B), we postulated that ELOVL4 inhibits other ELOVLs by hetero-oligomer interactions. To test this possibility, we performed coimmunoprecipitation analyses using anti-HA agarose beads, Myc-tagged ELOVL4, Myc-tagged ELOVL4ΔC, and HA-tagged ELOVLs. Although Myc-tagged wild-type ELOVL4 alone did not bind to anti-HA agarose (Figure 3B, lane 15), coexpression with one of the HA-tagged ELOVLs resulted in the recovery of Myc-tagged wild-type ELOVL4 in the immunoprecipitates, although its protein levels were low (Figure 3B, odd numbered lanes from 17 to 28). These results suggest that wild-type ELOVL4 interacts with all the ELOVLs, but only weakly. In contrast, the levels of Myc-ELOVL4ΔC in the immunoprecipitates of HA-ELOVLs were much higher than those of wild-type Myc-ELOVL4 (Figure 3B, even numbered lanes from 17 to 28), similar to interactions previously observed between ELOVL4 and ELOVL4ΔC (Figure 3A), suggesting strong interaction. These results indicate that ELOVL4ΔC forms not only homo-oligomers with wild-type ELOVL4 but also hetero-oligomers with other ELOVLs.

### ELOVL4ΔC interacts with KAR and TER

**Figure 4:**
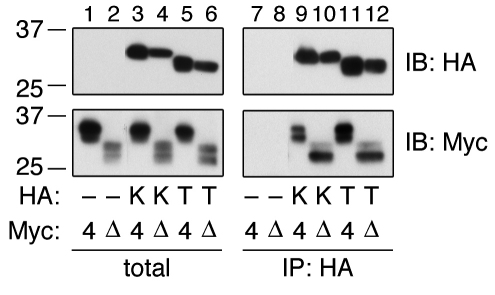
ELOVL4ΔC engages in the elongase complex. HEK 293T cells were transfected with a pCE-puro HA-1 (vector), pCE-puro HA-KAR, or pCE-puro HA-TER plasmid, and with the pCE-puro His_6_-Myc-ELOVL4 or pCE-puro His_6_-Myc-ELOVL4ΔC plasmid. Total cell lysates were prepared from the transfected cells and solubilized with 1% Triton X-100. Following immunoprecipitation with the anti-HA antibody, total lysates (1×) and bound proteins (IB: HA, 2×; IB: Myc, 5×) were subjected to immunoblotting with anti-HA or anti-Myc antibodies. IP, immunoprecipitation; IB, immunoblotting; -, vector; K, KAR; T, TER; 4, wild-type ELOVL4; Δ, ELOVL4ΔC.

VLCFA elongation occurs by cycling through a four-step process, i.e., condensation, reduction, dehydration, and reduction. We recently revealed that enzymes responsible for each step interact with each other and form an elongase complex [26]. To examine whether ELOVL4ΔC also engages in the elongase complex, we conducted coimmunoprecipitation analyses of the interactions between ELOVL4ΔC and the reductases KAR and TER, which catalyze the second and fourth steps of the VLCFA elongation cycle, respectively [12]. HA-tagged KAR or TER was expressed in HEK 293T cells, together with Myc-tagged ELOVL4 or ELOVL4ΔC. Wild-type Myc-ELOVL4 or Myc-ELOVL4ΔC alone did not bind to anti-HA agarose beads (Figure 4, lanes 7 and 8). However, wild-type Myc-ELOVL4 was detected in immunoprecipitates with HA-KAR (Figure 4, lane 9) or with HA-TER (lane 11), indicating that wild-type ELOVL4 interacts with KAR and TER. ELOVL4ΔC also interacted with both KAR and TER, similar to results with the wild-type protein (Figure 4, lanes 10 and 12). Thus, ELOVL4ΔC can form the elongase complex.

## Discussion

STGD3 is an autosomal dominant macular dystrophy and is caused by mutations in the *ELOVL4* gene [1,2]. ELOVL4 is an FA elongase whose substrates are considered to be fatty acyl-CoAs with ≥C26 carbon chain-length, identified using *Elovl4* knockout mice and *Elovl4*Δ*C* knockin mice [16-18]. Others had reported that forced expression of Elovl4 in cells treated with the FA EPA (C20:5(n-3)) or DPA (C22:5(n-3)) produced PUFAs with chain-lengths of C28 to C38 [23]. However, no direct biochemical analyses of ELOVL4 had been reported. We recently performed in vitro FA elongation assays using 11 different acyl-CoAs; these studies revealed that ELOVL4 is active only toward C24:0-CoA and C26:0-CoA [15]. Here, we likewise found that neither EPA nor DPA is a substrate of ELOVL4 (Figure 1B). We speculate that C22:5(n-3)-CoA is first elongated by ELOVL2, then is further elongated by ELOVL4 to acyl-CoAs having ≥26 carbon chain-lengths.

To date, the autosomal dominant transmission of STGD3 has been thought to be caused only by the interaction between wild-type ELOVL4 and ELOVL4ΔC. However, in retinas from *Elovl4*Δ*C* knockin mice, the FA composition is broadly affected [19], implying that Elovl4ΔC inhibits the entire VLCFA elongation machinery. In the present study we demonstrated that expression of ELOVL4ΔC results in the inhibition of elongation reactions toward C16:0-, C18:0-, C18:3(n-6)-, and C20:4(n-6)-CoAs (Figure 2B). Although these effects are weak, their long-term influence may cause multiple types of damage in cellular functions, leading to the STGD3 pathology. Indeed, development of STGD3 appears in humans around 14 years of age and in *Elovl4*Δ*C* knockin mice at several months [19,27]. Although in *Elovl4*Δ*C* knockin mice and STGD3 patients, appearance of symptoms occurs relatively long after birth, in our culture system the effects of ELOVL4ΔC expression on the elongation of several FAs were observed within two days. The differences in the onset of the effect may be due to differences in the expression levels of ELOVL4ΔC. In *Elovl4*Δ*C* knockin mice or STGD3 patients, ELOVL4ΔC must be expressed at levels equivalent to the allelic wild-type ELOVL4. In our cultured cell systems, however, ELOVL4ΔC is expressed at much higher levels.

Since elongation reactions toward C16:0-, C18:0-, C18:3(n-6)-, and C20:4(n-6)-CoAs are catalyzed by other ELOVLs, we had postulated the existence of hetero-oligomeric interactions between ELOVL4ΔC and the other ELOVLs. Although wild-type ELOVL4 interacted with other ELOVLs only weakly, substantial levels of ELOVL4ΔC did indeed interact with other ELOVLs (Figure 3B). An enhancement of the homo-oligomeric interaction was also observed between the C-terminally truncated ELOVL4 and wild-type ELOVL4 (Figure 3A). To date, 3 mutations have been found in the *ELOVL4* gene in STGD3 patients: a 5 bp deletion in exon 6 causing the loss of 51 amino acids at the C-terminus, a 2 bp deletion also leading to loss of the same 51 amino acids, and a nonsense mutation causing a 45 amino acid truncation [2]. Thus, all mutations cause a loss of at least 45 amino acid residues at the C-terminus; we used the 51 amino acid truncation mutant for the ELOVL4ΔC in this study. ELOVL4 is a multispan membrane protein and may have seven transmembrane segments, by analogy to its yeast homolog Sur4 [28]. The length of the C-terminal hydrophilic region (Arg268 to Asp314) is 47 amino acids. Therefore, the truncation of ELOVL4 observed in STGD3 causes complete or almost complete loss of the C-terminal hydrophilic region. Since this C-terminal region is the longest hydrophilic region in ELOVL4, it may contain residues important for function, protein–protein interaction, and maintenance of proper structure, in addition to the ER retention signal (Lys310-Ala311-Lys312-Gly313-Asp314) at the C-terminus. Although only deletion of the C-terminal ER retention signal has been emphasized in the pathology model of STGD3, we postulate that other residues in the C-terminal region may also be involved in this pathology. Loss of such residues may cause a conformational change in ELOVL4, leading to abnormal interactions with other ELOVLs.

In yeast, all components of VLCFA elongation form an elongase complex [28]. Mammalian enzymes also form one or more elongase complex(es). We previously revealed that the 3-hydroxyacyl-CoA dehydratase HACD proteins interact with ELOVLs, with some preferences [13]. HACD1 forms a complex not only with ELOVLs but also with the reductases KAR and TER [26]. In the present study, we demonstrated that ELOVL4 interacts with KAR and TER (Figure 4). Mammalian FA synthase is a multienzyme that incorporates all catalytic activities of its cyclic reaction, which is similar to VLCFA elongation, as discrete domains on a single polypeptide chain [29]. Like FA synthase, the adjacent positioning of the catalytic centers of the four elongation reactions of VLCFA elongation may contribute to efficient cycling.

ELOVL4ΔC interacts with other ELOVLs more strongly than the wild-type protein does, with apparently no specificity (Figure 3B). ELOVL4ΔC also interacts with KAR and TER, however in this case the strength of each interaction is comparable to that observed with the wild-type ELOVL4. Therefore, it is unlikely that ELOVL4ΔC binds to all proteins nonspecifically with a similar affinity. Nevertheless, we cannot exclude the possibility that ELOVL4ΔC also strongly interacts with other particular proteins and affects certain cellular functions. Since ELOVL4ΔC retains an ability to form the elongase complex, it is possible that ELOVL4ΔC competes with other ELOVLs when interacting with essential subunits of the elongase complex such as KAR and TER. In summary, we propose that several interactions, such as the already established ELOVL4-ELOVL4ΔC homo-oligomeric interaction, interactions with other ELOVLs, and interactions with other subunits of the elongase complex, contribute together to the pathology of STGD3.

## References

[1] Donoso LA Edwards AO Frost A Vrabec T Stone EM Hageman GS Perski T Autosomal dominant Stargardt-like macular dystrophy. Surv Ophthalmol 2001 46 149 63 11578648 10.1016/s0039-6257(01)00251-x

[2] Agbaga MP Mandal MN Anderson RE Retinal very long-chain PUFAs: new insights from studies on ELOVL4 protein. J Lipid Res 2010 51 1624 42 20299492 10.1194/jlr.R005025 PMC2882734

[3] Zhang K Kniazeva M Han M Li W Yu Z Yang Z Li Y Metzker ML Allikmets R Zack DJ Kakuk LE Lagali PS Wong PW MacDonald IM Sieving PA Figueroa DJ Austin CP Gould RJ Ayyagari R Petrukhin K A 5-bp deletion in *ELOVL4* is associated with two related forms of autosomal dominant macular dystrophy. Nat Genet 2001 27 89 93 11138005 10.1038/83817

[4] Mandal MN Ambasudhan R Wong PW Gage PJ Sieving PA Ayyagari R Characterization of mouse orthologue of *ELOVL4*: genomic organization and spatial and temporal expression. Genomics 2004 83 626 35 15028285 10.1016/j.ygeno.2003.09.020

[5] Ambasudhan R Wang X Jablonski MM Thompson DA Lagali PS Wong PW Sieving PA Ayyagari R Atrophic macular degeneration mutations in *ELOVL4* result in the intracellular misrouting of the protein. Genomics 2004 83 615 25 15028284 10.1016/j.ygeno.2003.10.004

[6] Vasireddy V Vijayasarathy C Huang J Wang XF Jablonski MM Petty HR Sieving PA Ayyagari R Stargardt-like macular dystrophy protein ELOVL4 exerts a dominant negative effect by recruiting wild-type protein into aggresomes. Mol Vis 2005 11 665 76 16163264

[7] Grayson C Molday RS Dominant negative mechanism underlies autosomal dominant Stargardt-like macular dystrophy linked to mutations in ELOVL4. J Biol Chem 2005 280 32521 30 16036915 10.1074/jbc.M503411200

[8] Karan G Yang Z Howes K Zhao Y Chen Y Cameron DJ Lin Y Pearson E Zhang K Loss of ER retention and sequestration of the wild-type ELOVL4 by Stargardt disease dominant negative mutants. Mol Vis 2005 11 657 64 16145543

[9] Spector AA Essentiality of fatty acids. Lipids 1999 34 Suppl S1 3 10419080 10.1007/BF02562220

[10] Leonard AE Pereira SL Sprecher H Huang YS Elongation of long-chain fatty acids. Prog Lipid Res 2004 43 36 54 14636670 10.1016/s0163-7827(03)00040-7

[11] Kihara A Sakuraba H Ikeda M Denpoh A Igarashi Y Membrane topology and essential amino acid residues of Phs1, a 3-hydroxyacyl-CoA dehydratase involved in very long-chain fatty acid elongation. J Biol Chem 2008 283 11199 209 18272525 10.1074/jbc.M708993200

[12] Moon YA Horton JD Identification of two mammalian reductases involved in the two-carbon fatty acyl elongation cascade. J Biol Chem 2003 278 7335 43 12482854 10.1074/jbc.M211684200

[13] Ikeda M Kanao Y Yamanaka M Sakuraba H Mizutani Y Igarashi Y Kihara A Characterization of four mammalian 3-hydroxyacyl-CoA dehydratases involved in very long-chain fatty acid synthesis. FEBS Lett 2008 582 2435 40 18554506 10.1016/j.febslet.2008.06.007

[14] Jakobsson A Westerberg R Jacobsson A Fatty acid elongases in mammals: their regulation and roles in metabolism. Prog Lipid Res 2006 45 237 49 16564093 10.1016/j.plipres.2006.01.004

[15] Ohno Y Suto S Yamanaka M Mizutani Y Mitsutake S Igarashi Y Sassa T Kihara A ELOVL1 production of C24 acyl-CoAs is linked to C24 sphingolipid synthesis. Proc Natl Acad Sci USA 2010 107 18439 44 20937905 10.1073/pnas.1005572107 PMC2973002

[16] Li W Sandhoff R Kono M Zerfas P Hoffmann V Ding BC Proia RL Deng CX Depletion of ceramides with very long chain fatty acids causes defective skin permeability barrier function, and neonatal lethality in ELOVL4 deficient mice. Int J Biol Sci 2007 3 120 8 17311087 10.7150/ijbs.3.120 PMC1796950

[17] McMahon A Butovich IA Mata NL Klein M Ritter R 3rd Richardson J Birch DG Edwards AO Kedzierski W Retinal pathology and skin barrier defect in mice carrying a Stargardt disease-3 mutation in elongase of very long chain fatty acids-4. Mol Vis 2007 13 258 72 17356513 PMC2633486

[18] Vasireddy V Uchida Y Salem N Jr Kim SY Mandal MN Reddy GB Bodepudi R Alderson NL Brown JC Hama H Dlugosz A Elias PM Holleran WM Ayyagari R Loss of functional ELOVL4 depletes very long-chain fatty acids (≥C28) and the unique ω-O-acylceramides in skin leading to neonatal death. Hum Mol Genet 2007 16 471 82 17208947 10.1093/hmg/ddl480 PMC1839956

[19] Vasireddy V Jablonski MM Mandal MN Raz-Prag D Wang XF Nizol L Iannaccone A Musch DC Bush RA Salem N Jr Sieving PA Ayyagari R *Elovl4* 5-bp-deletion knock-in mice develop progressive photoreceptor degeneration. Invest Ophthalmol Vis Sci 2006 47 4558 68 17003453 10.1167/iovs.06-0353

[20] McMahon A Jackson SN Woods AS Kedzierski W A Stargardt disease-3 mutation in the mouse Elovl4 gene causes retinal deficiency of C32–C36 acyl phosphatidylcholines. FEBS Lett 2007 581 5459 63 17983602 10.1016/j.febslet.2007.10.050 PMC2144913

[21] Moon YA Shah NA Mohapatra S Warrington JA Horton JD Identification of a mammalian long chain fatty acyl elongase regulated by sterol regulatory element-binding proteins. J Biol Chem 2001 276 45358 66 11567032 10.1074/jbc.M108413200

[22] Kihara A Ikeda M Kariya Y Lee EY Lee YM Igarashi Y Sphingosine-1-phosphate lyase is involved in the differentiation of F9 embryonal carcinoma cells to primitive endoderm. J Biol Chem 2003 278 14578 85 12584204 10.1074/jbc.M211416200

[23] Agbaga MP Brush RS Mandal MN Henry K Elliott MH Anderson RE Role of Stargardt-3 macular dystrophy protein (ELOVL4) in the biosynthesis of very long chain fatty acids. Proc Natl Acad Sci USA 2008 105 12843 8 18728184 10.1073/pnas.0802607105 PMC2525561

[24] Leonard AE Bobik EG Dorado J Kroeger PE Chuang LT Thurmond JM Parker-Barnes JM Das T Huang YS Mukerji P Cloning of a human cDNA encoding a novel enzyme involved in the elongation of long-chain polyunsaturated fatty acids. Biochem J 2000 350 765 70 10970790 PMC1221308

[25] Leonard AE Kelder B Bobik EG Chuang LT Lewis CJ Kopchick JJ Mukerji P Huang YS Identification and expression of mammalian long-chain PUFA elongation enzymes. Lipids 2002 37 733 40 12371743 10.1007/s11745-002-0955-6

[26] Konishi H Okuda A Ohno Y Kihara A Characterization of HACD1 K64Q mutant found in arrhythmogenic right ventricular dysplasia patients. J Biochem 2010 148 617 22 20724468 10.1093/jb/mvq092

[27] Edwards AO Miedziak A Vrabec T Verhoeven J Acott TS Weleber RG Donoso LA Autosomal dominant Stargardt-like macular dystrophy: I. Clinical characterization, longitudinal follow-up, and evidence for a common ancestry in families linked to chromosome 6q14. Am J Ophthalmol 1999 127 426 35 10218695 10.1016/s0002-9394(98)00331-6

[28] Denic V Weissman JS A molecular caliper mechanism for determining very long-chain fatty acid length. Cell 2007 130 663 77 17719544 10.1016/j.cell.2007.06.031

[29] Smith S The animal fatty acid synthase: one gene, one polypeptide, seven enzymes. FASEB J 1994 8 1248 59 8001737

